# Rayleigh anomaly-enabled mode hybridization in gold nanohole arrays by scalable colloidal lithography for highly-sensitive biosensing

**DOI:** 10.1515/nanoph-2021-0563

**Published:** 2022-01-12

**Authors:** Zhiliang Zhang, Feng Zhao, Renxian Gao, Chih-Yu Jao, Churong Ma, Jie Li, Xiangping Li, Bai-Ou Guan, Arif E. Cetin, Kai Chen

**Affiliations:** Guangdong Provincial Key Laboratory of Optical Fiber Sensing and Communications, Institute of Photonics Technology, Jinan University, Guangzhou, 511443, China; Department of Physics, Xiamen University, Xiamen, 361005, China; Institute of Advanced Wear & Corrosion Resistant and Functional Materials, Jinan University, Guangzhou, 510632, China; Izmir Biomedicine and Genome Center, Balcova 35340, Izmir, Turkey

**Keywords:** gold nanohole, Rayleigh anomaly, refractive index sensing, smartphone, surface plasmon

## Abstract

Plasmonic sensors exhibit tremendous potential to accomplish real-time, label-free, and high-sensitivity biosensing. Gold nanohole array (GNA) is one of the classic plasmonic nanostructures that can be readily fabricated and integrated into microfluidic platforms for a variety of applications. Even though GNA has been widely studied, new phenomena and applications are still emerging continuously expanding its capabilities. In this article, we demonstrated narrow-band high-order resonances enabled by Rayleigh anomaly in the nanohole arrays that are fabricated by scalable colloidal lithography. We fabricated large-area GNAs with different hole diameters, and investigated their transmission characteristics both numerically and experimentally. We showed that mode hybridization between the plasmon mode of the nanoholes and Rayleigh anomaly of the array could give rise to high-quality decapole resonance with a unique nearfield profile. We experimentally achieved a refractive index sensitivity, i.e., RIS up to 407 nm/RIU. More importantly, we introduced a spectrometer-free refractive index sensing based on lens-free smartphone imaging of GNAs with (intensity) sensitivity up to 137%/RIU. Using this platform, we realized the label-free detection of BSA molecules with concentration as low as 10^−8^ M. We believe our work could pave the way for highly sensitive and compact point-of-care devices with cost-effective and high-throughput plasmonic chips.

## 1 Introduction

Surface plasmon resonance (SPR), which originates from the collective oscillation of free electrons under an external excitation, has attracted significant attention due to its unprecedented capability of light manipulation and field enhancement in the nanoscale and has been utilized in a wide range of applications such as nonlinear optical devices [1], [2], [3], biological detection [4], [5], [6] and solar energy harvesting [7, 8]. SPR-based sensors are particularly promising as they could excite plasmon resonances that are highly sensitive to the variations within surrounding environment, which is critical for the realization of real-time, label-free, high-throughput and accurate sensing devices [9], [10], [11], [12], [13], [14], [15].

Among the various reported periodic nanostructures in literature, metallic nanohole arrays are one of the most investigated ones since the reporting of the extraordinary optical transmission (EOT) phenomenon by Ebbesen and his coworkers [16]. Gold nanohole array (GNA) based sensors have been employed in biomolecular sensing at various levels [17], [18], [19], [20], [21], [22], [23], [24], [25], [26]. For example, Gordon et al. [27] have demonstrated that GNAs could be employed to obtain molecular fingerprints via surface enhanced spectroscopy as well as environmental refractive index variations by monitoring the EOT resonance wavelength shift due to molecule absorptions. Introducing a high refractive index dielectric interlayer between GNAs and a transparent substrate, Cetin et al. [28] demonstrated isolated plasmonic modes, which avoids spectral overlap observed in conventional GNAs. Using this GNA based platform they demonstrated real-time monitoring of biomolecular binding interactions at sub-1 ng/mL levels. Introducing cavities underneath each nanohole have been also employed to improve the EOT properties and the sensing performance [29]. Although the majority of research in literature focuses on the sensing performance of the EOT resonance peaks (transmission maxima), transmission minima (or the spectral valleys) could exhibit higher refractive index sensitivities, which provides alternative routes to further improve the sensing characteristics of GNAs [30]. Furthermore, electric dipole modes of the nanoholes have been intensively studied, while in contrast, higher-order modes are rarely observed in nanoholes [31, 32], i.e., their properties and possible applications are needed to be investigated.

For commercial applications employing plasmonic sensors, one of the key issues is their portability and affordability. Up to now, bulky fiber spectrometers as the analytical device and halogen lamps as the light source are the “standard tools” in laboratory research that however cannot fulfill the requirements for practical applications [21, 27, 28, 33, 34]. This readout mechanism could be replaced with a camera-based plasmonic imaging platform. By integrating GNAs to a lens-free computational microscopy platform, detection of protein layer thickness down to ∼3 nm has been reported [35]. The findings by Cetin et al. demonstrated the feasibility of GNA-based biosensors in daily practice since smartphones are equipped with high-definition complementary metal oxide semiconductor (CMOS) cameras.

For the fabrication of GNAs, focused ion beam (FIB) and electron beam lithography (EBL) are the main tools because of their precise machining capability. However, their time-consuming and costly nature makes them unsuitable for large scale production and practicality [33, 35, 36]. On the other hand, colloidal lithography (CL), a scalable and versatile nanofabrication technique, could be used to fabricate large-area GNAs with low cost. But the relatively poor hole size distribution and defects in the arrays limits their sensing performance [30, 37], [38], [39]. Thus, improving the sensing performance of GNAs fabricated via CL could open doors for highly-sensitive biosensing based on GNAs.

In this work, we introduced a new mechanism to improve the sensing capability of GNAs by exciting high-order modes with narrow bandwidth enabled by the hybridization of LSPRs of individual nanoholes and Rayleigh anomaly of the array. We fabricated hexagonal GNAs on SiO_2_ substrate with CL using reactive ion etching (RIE) and metal deposition. We characterized the GNA transmission spectra with a fiber spectrometer, and analyzed the plasmon modes through numerical simulations. By reducing O_2_ plasma etching time, we intend to create nanoholes with larger diameter that almost conjoin with the adjacent nanoholes and thus effectively overlap their LSPRs with the Rayleigh anomaly. Such mode coupling gives rise to a narrow-band decapole resonant mode that exhibits large refractive index sensitivities (RIS), as large as, 456 nm/RIU in simulation and 407 nm/RIU in experiment, which is much higher than that of the corresponding EOT wavelength of the same GNA. Furthermore, we took advantage of the high sensitivity of this hybrid mode and developed a smartphone-based biosensor employing a light-emitting-diode (LED) source. Utilizing the data from the red (R) channel of the CMOS camera, we determined a relative intensity change, as large as, 137%/RIU, which could be very advantageous for biosensing applications in field settings. In order to demonstrate the sensing capabilities of our portable GNA-based sensor, we performed the label-free detection of bovine serum albumin (BSA). A BSA concentration down to 10^−8^ M can be detected using a fiber spectrometer and the smartphone-based sensing platforms. Smartphones, equipped with high-definition complementary metal oxide semiconductor (CMOS) camera, could provide an alternative readout mechanism for biosensing applications in daily practice.

## 2 Results and discussions

**Figure 1:**
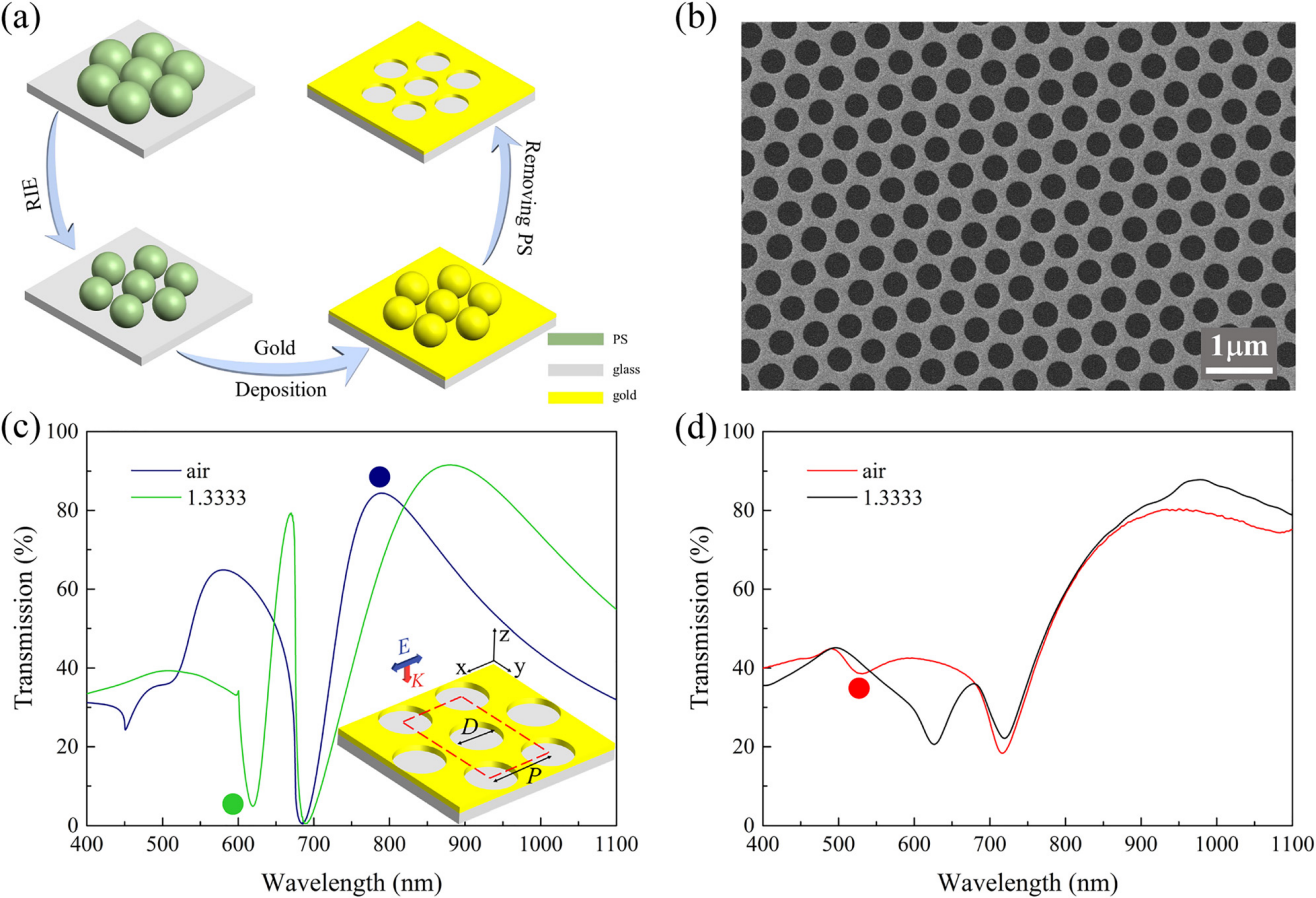
Fabrication and optical characterization of GNAs. (a) Schematic of the fabrication process of GNA. (b) A representative SEM image of the nanohole arrays (*D* = 350 nm). The simulated (c) and experimental (d) transmission spectra of the GNA (*p* = 520 nm, *D* = 350 nm) in air (*n* = 1) and water (*n* = 1.3333). Inset in Figure 3c: geometrical configuration of the GNA. *D* represents the diameter of the nanohole, and *P* represents the period of the array. *E* and *k* are the polarization and propagation directions of the incident light source. The color dots denote spectra features of peak at 789 nm (blue dot), dip at 619 nm (green dot) in (c) and a shallow dip at 531 nm (red dot) in (d).

The GNAs are fabricated on SiO_2_ substrates with a multistep process based on CL as illustrated in Figure 1a. First, a close-packed monolayer of polystyrene (PS) nanospheres are formed on SiO_2_ substrate through self-assembly of the nanospheres as reported in our previous work [40, 41]. Then the nanospheres are subjected to O_2_ plasma treatment with RIE. Finally, with the etched nanosphere arrays as masks, 70 nm gold (3 nm Cr as adhesion layer) is deposited on the substrate followed by removal of the PS nanospheres with ultrasonication in toluene forming GNAs on SiO_2_ with a fixed period and tunable nanohole diameters. Figure 1b shows a scanning electron microscopy (SEM) image of the fabricated GNA with a period of 520 nm and hole diameter of 350 nm. The nanohole size as well as the array periodicity is quite uniform over a large area, which suggests good quality of the nanosphere monolayers. Simulated and measured transmission spectra of the GNA in air (blue) and water (green) are depicted in Figure 1c and d, respectively. The inset in Figure 1c illustrates the geometrical configuration of the GNA and the unit cell (red dashed line) used in the simulations. Figure 1c and d shows the good correlation between experiments and simulations except for some minor deviations in peak/dip position and intensity, which could be attributed to the imperfectness and inhomogeneity of the GNAs prepared with CL.

**Figure 2:**
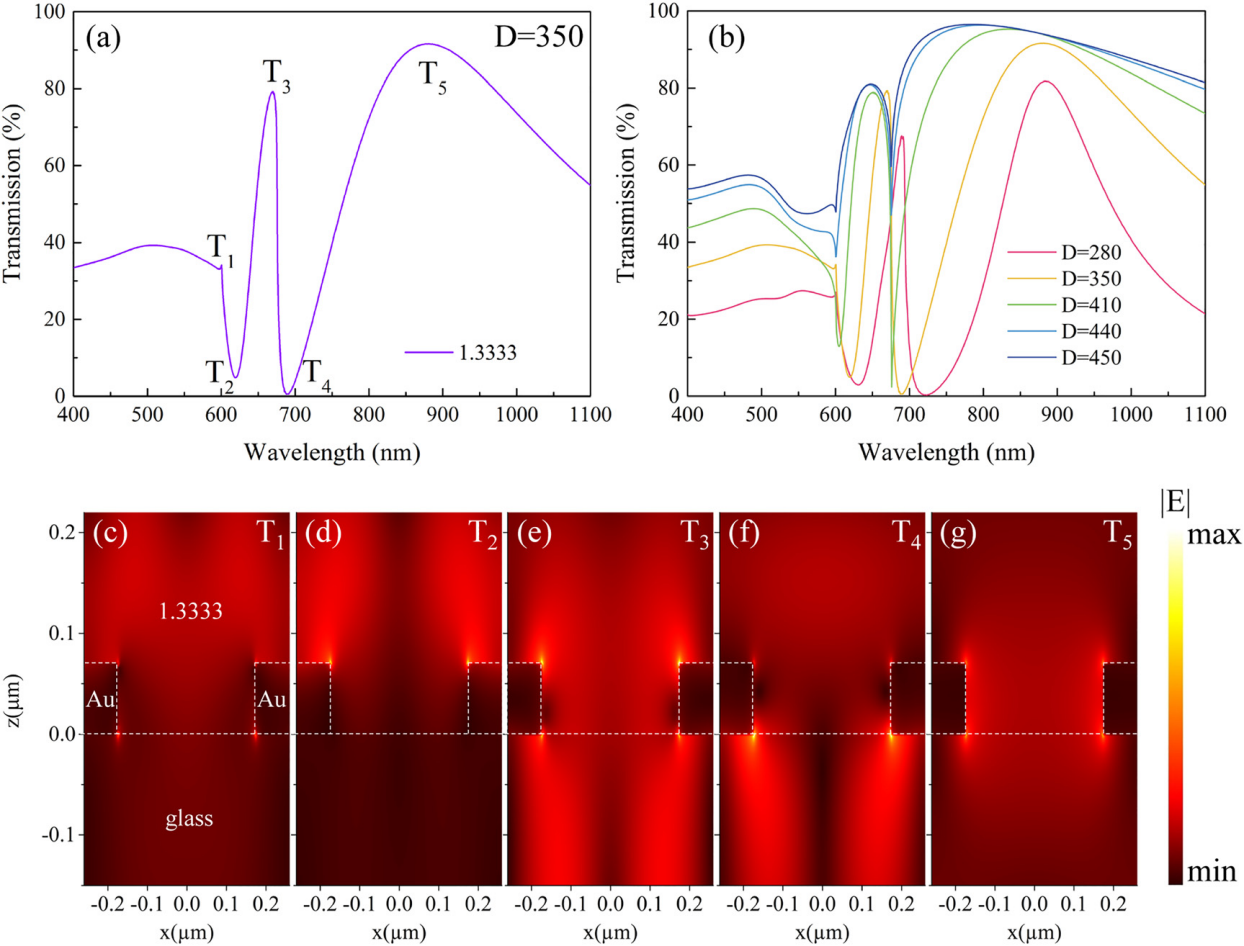
The simulation results of the far field and near field properties of GNAs. (**a**) Simulated spectra of the GNA (*p* = 520 nm, *D* = 350 nm). (**b**) The transmission spectra of GNAs with different diameters from 280 to 450 nm. (**c**), (**d**), (**e**), (**f**), (**g**): E-field amplitude distribution of T_1_ to T_5_, respectively.

As shown in Figure 1c, the transmission spectrum exhibits dramatic changes when the environmental refractive index (*n*) varies from 1 (air) to 1.3333 (water). While the major peak at 789 nm (denoted with a blue dot) shows a red-shift, a much narrower dip emerges at 619 nm (denoted with a green dot) on the spectrum for water. The experimental results in Figure 1d show similar spectral variations, where the new spectral dip in water evolves from a shallow resonance dip at 531 nm in air (denoted with a red dot). It has been noted that resonance peaks and dips supported by nanohole arrays could exhibit different responses to the external refractive index changes [33]. Therefore, we numerically studied the mode properties of the spectral peaks and dips observed in the transmission spectrum of the GNA in water. Figure 2a shows the simulated transmission spectrum of GNA with diameter *D* = 350 nm, where the background refractive index is 1.3333. In the figure, the transmission extrema are labeled with T_1_ to T_5_. The electric nearfield distribution of the five modes in *x*–*z* plane is displayed in Figure 2c–g. For T_1_ and T_2_, the enhanced electric fields mainly locate at the top surface of the GNA extensively extends into the medium. Electric field corresponding to T_3_ locates inside the aperture, and exhibits a similar intensity distribution on both sides of the gold surface with “hot spots” at the top edges of the aperture. For T_4_, the electric field within the medium becomes weaker and the “hot spots” are observed on the bottom edges of the aperture. As shown in Figure 2g, electric field enhancement for T_5_ is the weakest and is concentrated mainly near the inner side of the nanohole.

The electric nearfield profile at the Au/medium and Au/glass interfaces could help to characterize the nature of different modes, and to evaluate their corresponding sensitivities to environmental refractive index changes. Comparing the calculated wavelengths of the specific surface modes with the simulated transmission spectra we can assign T5 mode as the Au/glass (1, 0) SPP mode, while T3 mode as the Au/water (1, 0) SPP mode. Similarly, T1 and T4 modes can be assigned to the Au/water (1, 0) and Au/glass (1, 0) Rayleigh Anomaly mode. For T2 mode, by investigating its near-field distribution and contemplating related references [42], [43], [44], T2 mode is assigned as the LSPR mode of the nanohole (see Supplementary information for details). In designing plasmonic nanoantennas, large field enhancement is generally desired. To improve RI sensitivities, it is very critical that these local electromagnetic fields with high intensity enhancement are accessible to the medium adjacent to the plasmonic structures of interest. For T_3_, T_4_, and T_5_, the presence of the supporting substrate occupies majority of the enhanced field volume, i.e., they are not accessible to the medium. Among the five modes supported by GNAs, T_2_ mode provides the largest field enhancement as well as the largest available sensing volume, which makes it very advantageous for label-free biosensing. We demonstrated such advantage of T_2_ over the other modes through simulated sensing performances of different modes as shown in Supplementary Figure S1, where T_2_ shows the largest RI sensitivity.

To obtain a better understanding of the high sensitivity of T_2_ mode, further investigation was carried out on the effect of nanohole diameter on the T_2_ sensing characteristics. Transmission spectra of GNAs in water (*n* = 1.3333) with *D* = 280, 350, 410, 440, and 450 nm are shown in Figure 2b. As the nanohole diameter increases, the resonance wavelength of T_2_ mode experiences a significant blue-shift accompanied by a progressively narrowing bandwidth. For example, When the hole diameter increases from *D* = 280 to 410 nm, bandwidth of T_2_ reduces from 43.2 to 9.3 nm. With *D* = 440 nm, the resonance wavelength of T_2_ mode overlaps with that of T_1_ mode, which results in a coupled plasmon mode with a ultra-narrow bandwidth of 2.7 nm.

**Figure 3:**
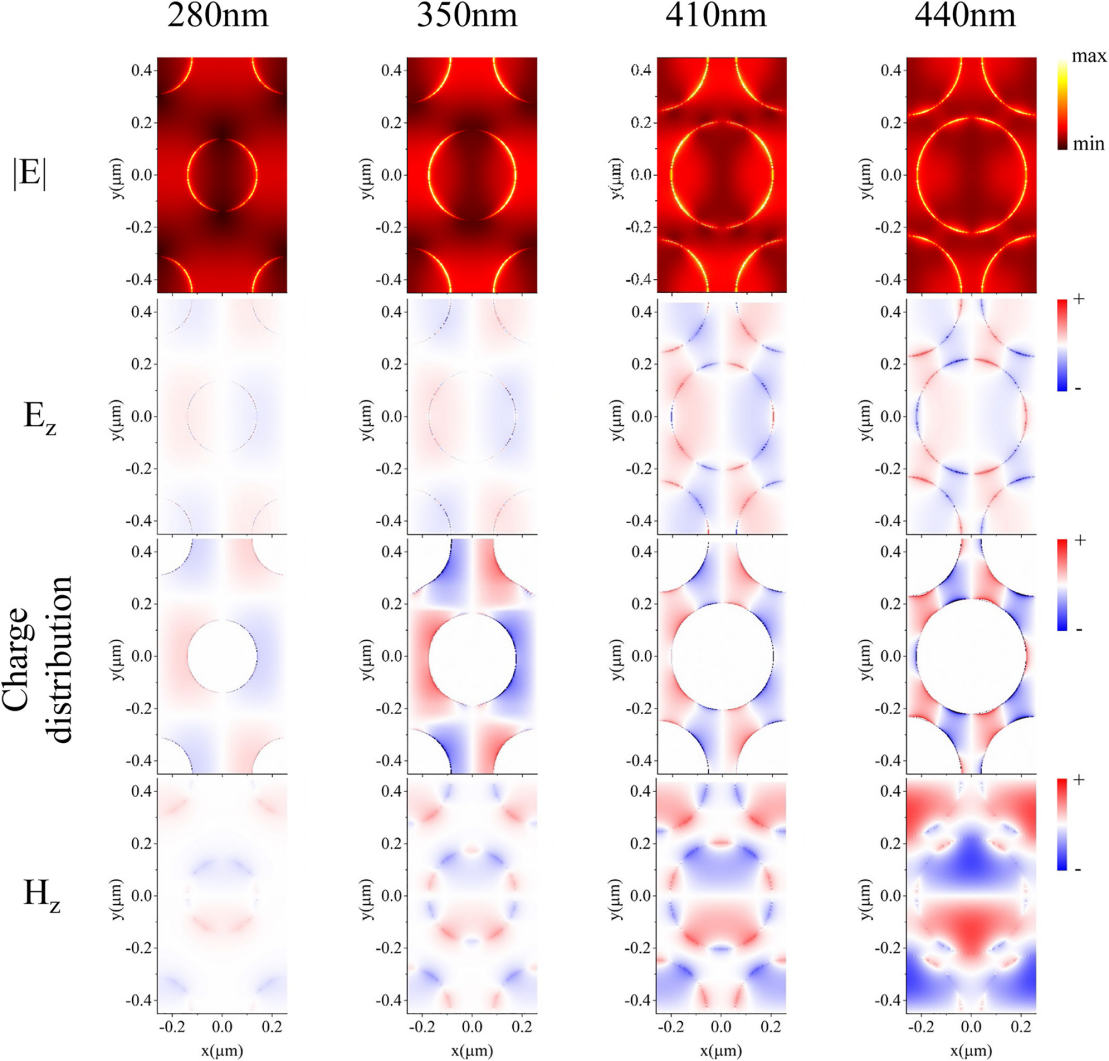
Distribution of electric field amplitude, **E**_**z**_ component, charge distribution and **H**_**z**_ component in the *x*–*y* plane (*z* = 0) with diameters of 280, 350, 410, and 440 nm.

Figure 3 shows the electric field amplitude **E**, *z* components of the electric field and magnetic field (**E**_**z**_, **H**_**z**_), and the charge distribution in the *x*–*y* plane (glass-gold interface) of GNAs with *D* = 280, 350, 410, and 440 nm for T_2_. As can be seen from the first row of Figure 3, the electric field enhancement gradually becomes larger when the hole diameter increases from 280 to 440 nm, while its distribution pattern evolves from dipole-like (280 nm, 350 nm) to multipole-like (410 nm, 440 nm) indicating a continuous mode coupling process of T_1_ and T_2_. As the hole diameter increases, T_1_ and T_2_ grow into an intensely coupled hybrid mode, which is the origin of the ultra-narrow bandwidth at *D* = 440 nm. This mode evolution with hole diameters could be also seen with the **E**_**z**_, **H**_**z**_ profile and the charge distribution. For *D* = 280 and 350 nm, **E**_**z**_ and the charge distribution exhibit a dipole-like pattern, while for *D* = 410 and 440 nm, the distribution turns into a decapole pattern, suggesting the occurrence of the progressive coupling between T_1_ and T_2_ modes. Although plasmonic nanoholes have been widely studied, high-order modes are rarely observed and discussed, particularly for nanohole arrays fabricated by colloidal lithography. Therefore, it is of great interest and importance to investigate the sensing properties of such high-order narrow-band modes inside the large-area nanohole arrays.

**Figure 4:**
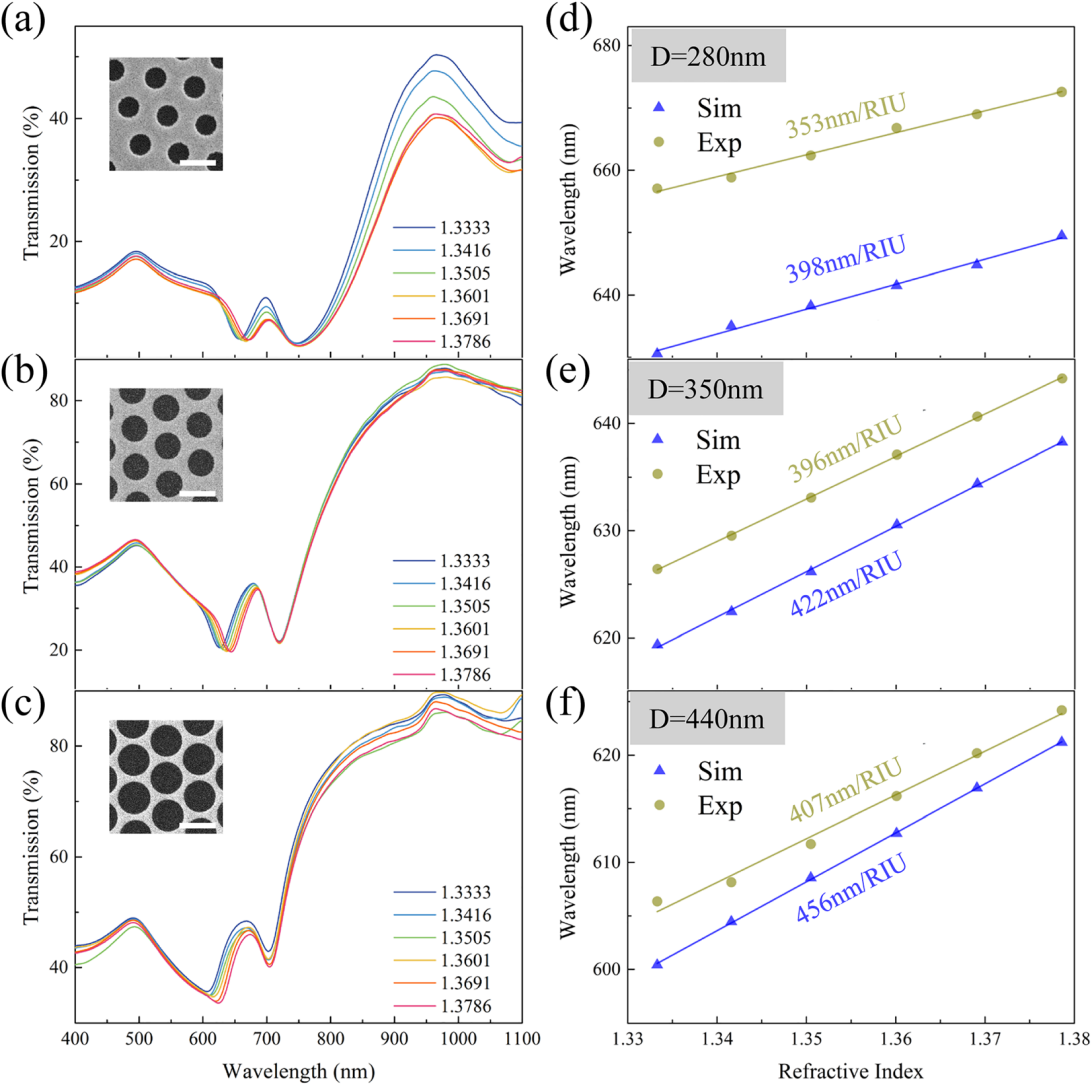
The RI sensing performance of the T_2_ mode in nanohole arrays. The transmission spectra of GNAs with (a) *D* = 280 nm, (b) 350 nm, and (c) 440 nm within different refractive indices from 1.3333 to 1.3786. The inset images in a–c: SEM graphs of the GNA measured. The scale bar in all SEM images is 500 nm. The simulated and experimental sensitivity of the resonance wavelength of T_1_/T_2_ strongly coupling mode from GNA with (d) *D* = 280 nm, (e) 350 nm, and (f) 440 nm.

Sensing performance of the T_2_ mode was experimentally and numerically investigated. Transmission spectra of GNAs with *D* = 280, 350 and 440 nm were simulated and measured while varying the environmental refractive index from 1.3333 (water) to 1.3786. The sensitivity of GNAs, *S*, is defined as the equation below, which is calculated from the slope of the linear relation between Δλ and Δn :

$$s=\frac{\Delta \lambda }{\Delta n}$$

where Δλ is the wavelength shift of T_2_ mode, and Δn is the corresponding change of the refractive index of the adjacent media. Figure 4a–c shows the experimental transmission spectra of GNA with *D* = 280, 350 and 440 nm, respectively, for different refractive indices (see Supplementary Figure S2 for the simulated spectra). In experiments, refractive index of the medium was adjusted by immersing GNAs into NaCl solutions with different concentrations, where the refractive index of each solution was determined by a refractometer. For each GNA, the resonance dip (T_2_ mode) shows continuous red-shift with increasing environmental refractive index as expected. When the environmental refractive index is the same, the resonance wavelength shows a blue-shift while the corresponding bandwidth becomes narrower as the nanohole diameter increases, which is consistent with the simulation shown in Figure 2b. Although the spectral dip appears wider in Figure 4c, its asymmetric shape indicates that the dip is actually a combination of a broad spectral dip at shorter wavelength and a narrow spectral dip at longer wavelength, which is again in good agreement with the simulation in Figure 2b that for *D* = 440 nm, a broad T_2_ mode moves past the Rayleigh anomaly of T_1_ to its blue side. The simulated and experimental sensitivity of the GNAs with three different diameters are shown in Figure 4d (*D* = 280 nm), 4e (*D* = 350 nm), and 4f (*D* = 440 nm). By utilizing the hybrid mode of T_1_ and T_2_, the largest sensitivity of 407 and 456 nm/RIU can be obtained in experiment and simulation, respectively with *D* = 440 nm. The sensing performance of T_2_ mode proves our analyses in Figure 2 that large and accessible fields supported by narrow plasmonic modes afford better sensing performance, which arises through the hybridization of two plasmonic modes in our system. Here, the simulated data shows larger sensitivities than the experimental ones, which could be attributed to the imperfectness and inhomogeneity in the fabricated GNAs arising from the size distribution of the PS spheres and the defects in the sphere monolayers.

**Figure 5:**
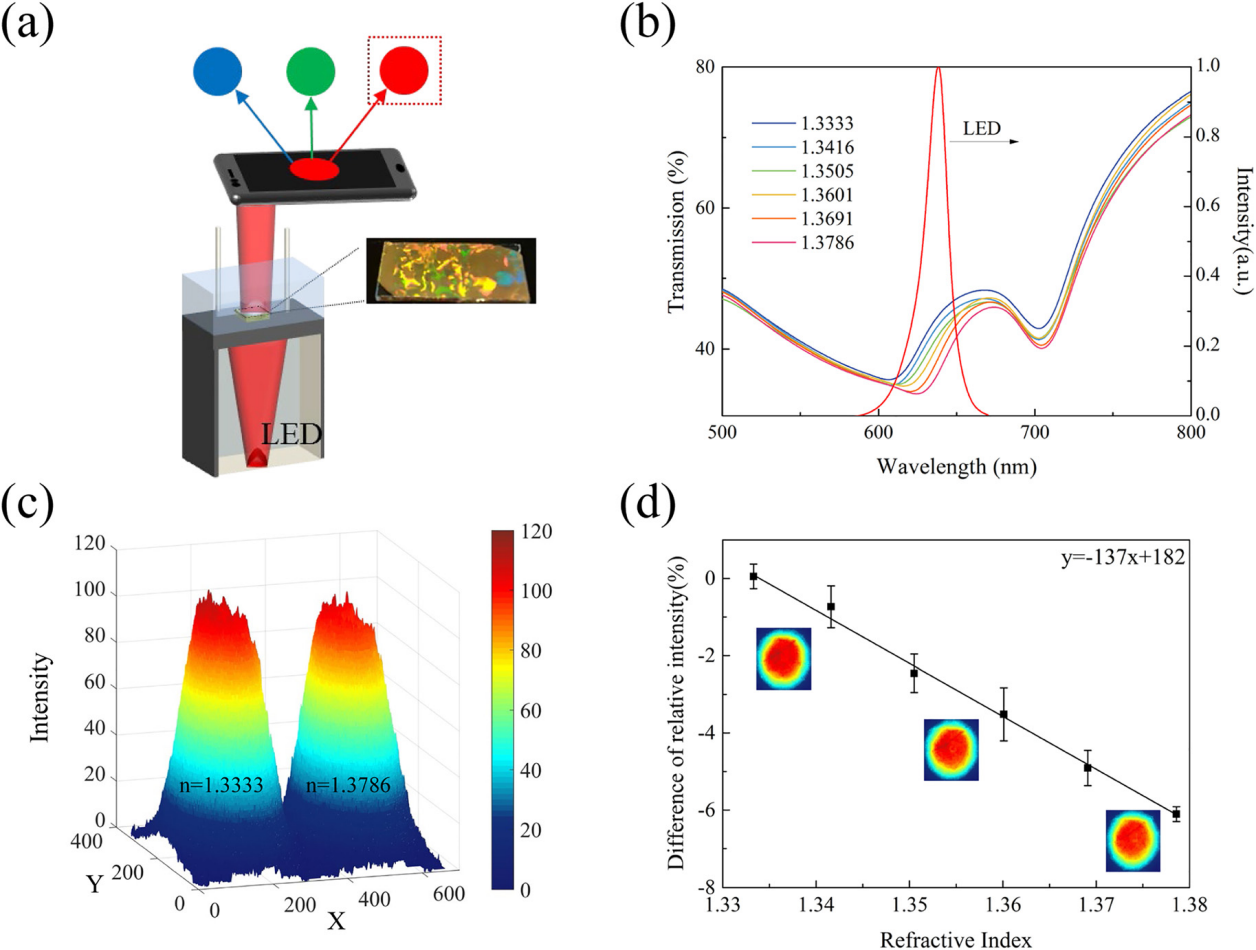
The sensing performance of the smartphone-based sensing platform. (**a**) Schematic illustration of the sensing platform. (**b**) Transmission spectra of GNAs (*D* = 440 nm) and the luminescent spectrum of LED. (**c**) R channel values obtained from GNAs in water and NaCl solution (*n* = 1.3786) by our sensing platform. (**d**) Calculated difference of relative intensity in water and different concentration of NaCl solutions. Inset images show the *R* value intensity distribution in water, NaCl solution with *n* = 1.3505 and NaCl solution with *n* = 1.3786 (top to bottom).

In order to meet the requirements of portability and cost-effectiveness in practical applications, spectrometer-free setup is crucial for the development of future compact sensing devices. Smartphones have the potential to be utilized as a spectroscopic tool as they have a built-in CMOS camera that could monitor incident light intensity by imaging. Using a smartphone camera as a photodetector and its RGB channels as optical filters, the schematic of the proposed biosensing platform is shown in Figure 5a. The GNA sample is integrated into a microfluidic channel and an LED source is positioned inside a sealed box with a small aperture (diameter = 1 mm) to eliminate strayed light. Through this aperture, light out from this box could be easily captured by the camera after passing through the plasmonic chip.

To yield the highest sensitivity, we used the GNA chip with *D* = 440 nm. Figure 5b shows the transmission spectra of the sample for refractive indices from 1.3333 to 1.3786, and the emission spectrum of the LED source. As shown in Figure 4c and b, the right side of the resonance dip (the hybrid mode) shows a steady red-shift in the range between 600 and 680 nm as the medium refractive index increases from 1.3333 to 1.3786. Thus, we chose an LED source with a central wavelength of 638 nm, which ensures a good overlap between the source and the linear range of the spectral variations within the GNA transmission spectra enabling an intensity-based imaging method for RI sensing using smartphone camera. To illustrate the working principle of our sensing platform, the intensity data from the R channel were extracted from the photo images of the sensor chip in water (*n* = 1.3333) and NaCl solution (*n* = 1.3786) as shown in Figure 5c. As the transmission spectrum better overlaps with that of LED for lower refractive indices (i.e., larger transmission values fall into the LED spectrum enabling more photons reaching the CMOS active area), *R* value calculated for water is higher than NaCl solution. Using the *R* value for water as reference, we calculated the difference of the relative intensity for different refractive indices and plotted the data in Figure 5d. The sensitivity of the smartphone-based GNA sensor (calculated from the slope of the linear fitting curve in Figure 5d) is obtained as 137%/RIU, which is a very advantageous value for label-free biosensing applications in resource-poor settings.

**Figure 6:**
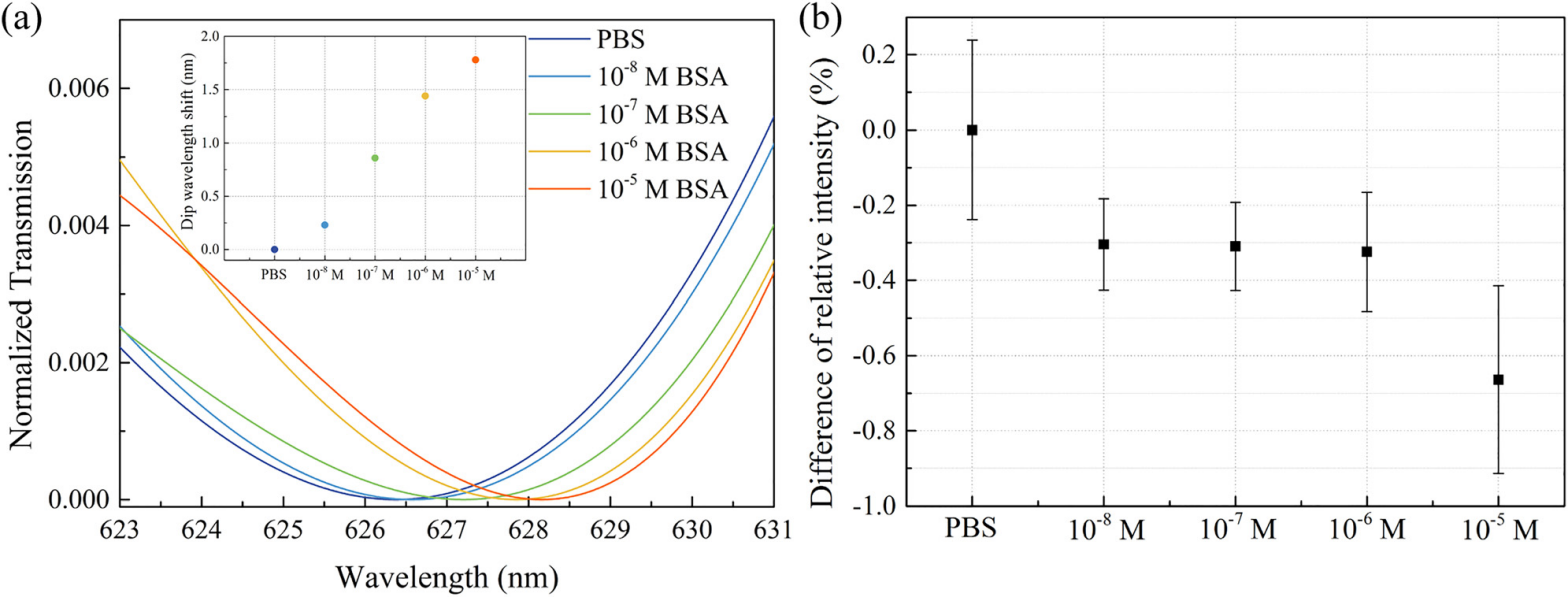
Biosensing of BSA molecules with the GNA sensors (*D* = 440 nm) (a) by monitoring the resonance wavelength shift using a fiber spectrometer and (b) by measuring the intensity change of the transmitted LED light using a smartphone camera. The transmission spectra in (a) are the results of polynomial fitting of the original spectra. The inset in (a) shows the linear relationship between the resonance wavelength shift and the BSA concentration.

In order to demonstrate the practicality of our portable biosensor, we used BSA as a model analyte and performed its label-free detection. Figure 6a shows the transmission spectra for BSA concentrations between 10^−8^ and 10^−5^ M. Here, we used a third-order polynomial fitting to extract the resonance wavelengths. We observed a linear relationship between the BSA concentration and the resonance wavelength shift, where we could detect BSA concentration as low as 10^−8^ M. For the smartphone-based sensing platform, difference of relative intensity (Figure 6b) shows a decreasing trend with increasing BSA concentration. Compared with the spectrometer-based readout shown in Figure 6a, the data determined from the smartphone-based platform exhibits more deviations, which could be attributed to the instability of the LED source and the smartphone camera. Nevertheless, these results confirm that our plasmonic smartphone-based sensor is highly promising for low-cost detection of biomolecules for field-settings.

In summary, we investigated the narrow-band decapole resonance modes in gold nanohole arrays fabricated by scalable colloidal lithography. We showed that this rarely observed high-order mode could be readily excited via the coupling between the LSPR modes of the nanoholes and the Rayleigh anomaly of the array. The high quality of this resonance associated with highly accessible large local electromagnetic fields offers high refractive index sensitivity. We also introduced a smartphone-based biosensing setup employing the relative change of the transmission intensities and achieved a sensitivity as large as 137%/RIU. We could successfully detect BSA molecules with concentrations as low as 10^−8^ M with both spectrometer- and smartphone-based readout. We believe the unique far- and nearfield properties of high-order modes of plasmonic nanohole arrays fabricated through a simple fabrication method over a large area, could open doors for a sensitive and high-throughput biosensing platform for resource-poor settings.

## 3 Experiments and methods

### 3.1 Nanohole fabrication

Sulfate-functionalized polystyrene spheres with nominal diameter of 500 nm were purchased from Polyscience Inc. and dispersed in a water/ethanol mixture (1:1 volume ratio). Glass substrates were cleaned in a mixture of NH_4_OH/H_2_O_2_/H_2_O (1:1:5 volume ratio) at 80 °C for 15 min and blown dry with N_2_. The nanospheres formed a close-packed monolayer on the glass substrates by colloidal lithography method reported previously [40]. Diameter of the PS spheres was reduced by O_2_ plasma with reactive ion etching (RIE). The etching parameters in our fabrication process were set as the following: the oxygen flow was 100 sccm, the pressure of the chamber was 376 mTorr, the RF power was 100 W and the etching time was 60 s (*D* = 440 nm), 80 s (*D* = 350 nm) and 85 s (*D* = 280 nm), respectively. 70 nm Au was deposited with 3 nm Cr as adhesion layer with electron beam deposition. PS nanospheres were removed with sonication in ethanol for 5 min.

### 3.2 Numerical simulation

Finite-domain-time-difference (FDTD) method was used to simulate the far field and near field properties of the nanohole arrays. In simulations, periodic boundary conditions were used in *x* and *y* directions, and perfectly matched layer (PML) boundary condition was used in *z* direction. Refractive index of the glass substrate was fixed at 1.5, and the dielectric constant of Au were taken from Johnson and Christy [45].

### 3.3 Refractive index sensing

NaCl solutions were prepared by dissolving NaCl in deionized (DI) water with different concentrations, yielding refractive indices from 1.3416 to 1.3786 determined with a refractometer (Hangzhou Lohand Biological Technology Co., Ltd.). Transmission spectra of GNAs were measured by a fiber-coupled spectrometer (Ocean Optics). A broadband halogen lamp was used as the light source. In each experiment, plasmonic chip was positioned in a standard microfluidic channel.

For the smartphone-based sensing, an LED source (Thorlabs, Inc.) is used as the light source. The smartphone in our experiment is Xiaomi Note3, which includes a 16-megapixel camera. In the manual mode, the imaging parameters were set to white balance 5600, shuttering time 1/1000 s and International Standards Organization (ISO) Light Sensibility Ordinance 100. Five images were taken for each NaCl solutions with different concentrations. We then extracted and calculated the average of the intensity values of the R channel using MATLAB.

### 3.4 Analyte preparation

Bovine serum albumin (5%, Solarbio, Beijing) was diluted in PBS (Solarbio, Beijing) with different concentrations. The experiments were done by injecting BSA solutions (from low concentration of 10^−8^ M to high concentration of 10^−5^ M) into the microfluidic chamber containing the plasmonic chip. We then measured the wavelength shift of the reflectance spectra with the spectrometer and the image intensities with the smartphone camera after an identical reaction time. Afterwards, a new BSA solution of higher concentration was injected for the following measurement.

## Supplementary Material

Supplementary Material
