# Supplementary material for: Rayleigh anomaly-enabled mode hybridization in gold nanohole arrays by scalable colloidal lithography for highly-sensitive biosensing
Source: Nanophotonics. 2022 Jan 12;11(3):507–17. doi: 10.1515/nanoph-2021-0563 (PMC11502056; doi:10.1515/nanoph-2021-0563)
Supplement: Supplementary file 1 — Supplementary Material [file j_nanoph-2021-0563_suppl.docx]

**Supporting Information**

Rayleigh Anomaly-enabled Mode Hybridization in Gold Nanohole Arrays by Scalable Colloidal Lithography for Ultra-sensitive Biosensing

Zhiliang Zhang^1, #^, Feng Zhao^1, #^, Renxian Gao^2^, Chih-Yu Jao^3^, Churong Ma^1^, Jie Li^1^, Xiangping Li^1^, Bai-Ou Guan^1^, Arif E. Cetin^4^, Kai Chen^1^*

^1^Guangdong Provincial Key Laboratory of Optical Fiber Sensing and Communications, Institute of Photonics Technology, Jinan University, Guangzhou, 511443, China

^2^Department of Physics, Xiamen University, Xiamen, 361005, China

^3^Institute of Advanced Wear & Corrosion Resistant and Functional Materials, Jinan University, Guangzhou, 510632, China

^4^Izmir Biomedicine and Genome Center, Balcova 35340, Izmir, Turkey

1. **Mode calculations in the GNAs**

For nanohole arrays with a hexagonal configuration, transmission peaks and dips can be analyzed according to the following equations^[1-3]^:

$$\begin{aligned} \lambda_{max}\left( i,j \right)= \frac{\sqrt{3}}{2}\frac{P}{\sqrt{i^{2}+{ij+j}^{2}}}\sqrt{\frac{\varepsilon_{1}\varepsilon_{d}}{\varepsilon_{1}+\varepsilon_{d}}}, \end{aligned}$$

$$\begin{aligned} \lambda_{min}\left( i,j \right)= \frac{\sqrt{3}}{2}\frac{P}{\sqrt{i^{2}+{ij+j}^{2}}}\sqrt{\varepsilon_{d}} , \end{aligned}$$

where $\lambda_{max}(i,j)$ is the transmission peak position, $\lambda_{min}(i,j)$ is the transmission dip position, $P$ is the period of nanohole arrays, $i$ and $j$ are integer indexes of the peaks/dips, and $\varepsilon_{1}$and $\varepsilon_{d}$ are the real part of the relative permittivity of the metal film and the surrounding environment. According to these two equations, for transmission peak the Au/glass (1, 0) SPP mode was at 722 nm, Au/water (1, 0) SPP mode was at 646 nm, while Au/glass (1, 1) SPP mode at 533 nm and Au/water (1, 1) SPP mode at 498 nm, while for transmission dip, the Au/glass (1, 0) RA mode at 675 nm and Au/water (1, 0) RA mode at 600 nm. Combining the calculated wavelength of specific surface modes with the simulated transmission spectra (please see Figure R4), we can assign the T5 mode as the Au/glass (1, 0) SPP mode, while T3 as the Au/water (1, 0) SPP mode. Similarly, the T1 and T4 can be assign to the Au/water (1, 0) and Au/glass (1, 0) Ranyleigh Anomaly mode. For T2 mode, by investigating its near-field distribution and contemplating related references^[4-6]^, T2 mode is assigned as the LSPR mode of the nanohole.

1. **FDTD simulations of the near-field response and sensing performance of different modes in the nanohole arrays**

Fig. S1 shows the simulated near-field response and sensing performance of the different modes from the hexagonal Au-nanohole arrays. As shown in Figure S1a, with the environmental refractive index increasing, the resonance wavelength shifts of T_3_ and T_4_ modes are quite small. By contrast, the resonance wavelength of T_2_ mode and T_5_ mode exhibits a significant red shift. Fig. S1b shows that the sensitivity of T_2_ to T_5_ modes is 422 nm/RIU, 128 nm/RIU, 0 nm/RIU and 375 nm/RIU, respectively. The simulation results indicate that T_2_ mode is the best choice for refractive index sensing among the modes.

The near field profiles of T_2_ and T_5_ modes were simulated and the electric filed distribution in the x-y plane (z=0) and x-z plane (y=0) was displayed in Fig. S1c-f, respectively. The enhanced electric field intensity of the T_2_ mode are around the top nanohole rims (Fig. S1c, e) while for T_5_ mode the enhancement of near field mostly concentrates inside the nanohole.


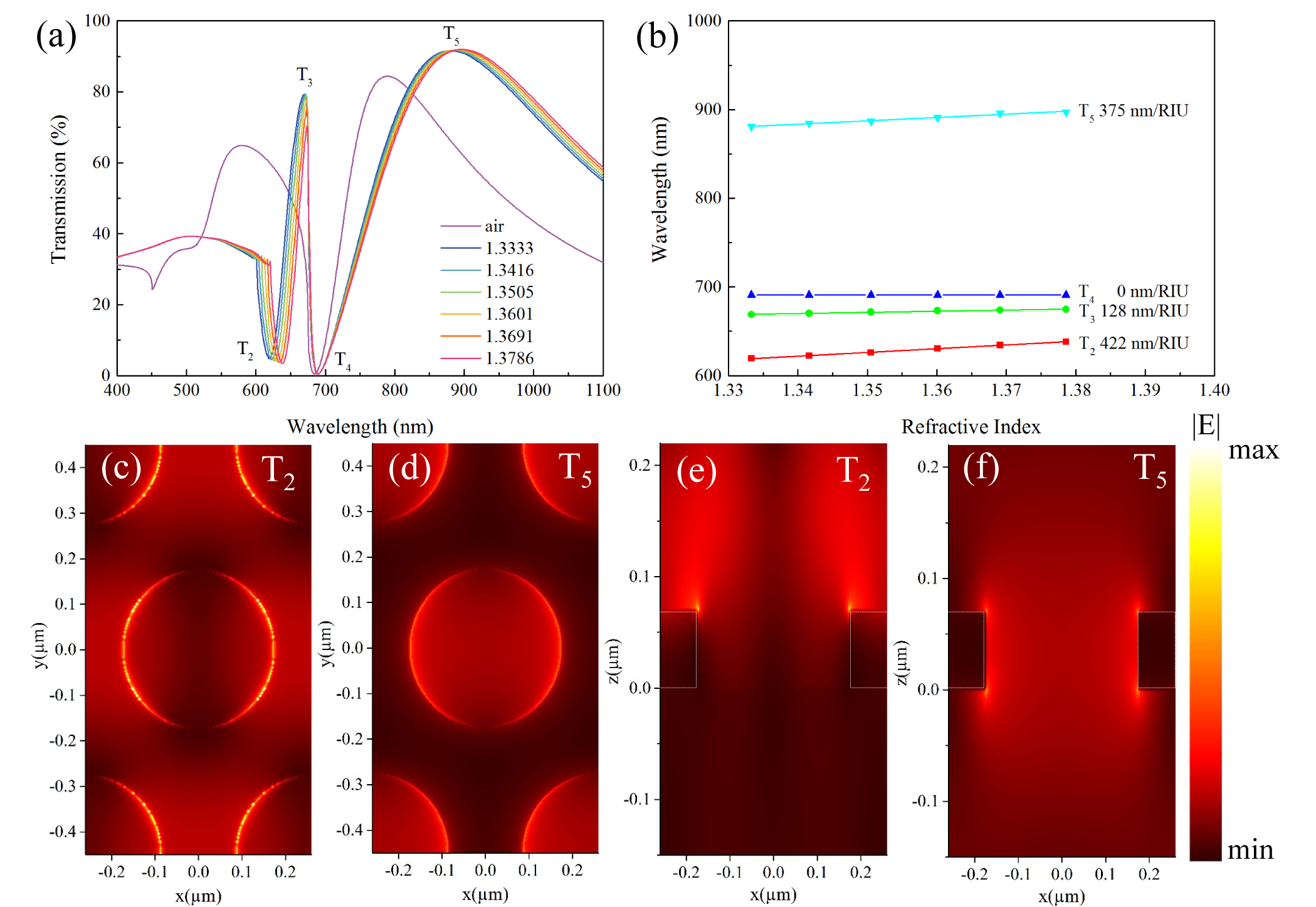


**Fig. S1.** The simulation results of near-field distribution and sensing performance of the different modes in the nanohole array (P=520nm, D=350nm). (**a**) The simulated transmission spectra of the nanohole array in air and different media with refractive index from 1.3333 to 1.3786. (**b**) The sensing sensitivity of T_2_, T_3_, T_4_ and T_5_ modes. Electric field distribution of T_2_ mode (**c**) and T_5_ mode (**d**) in x-y plane (z=0) and the electric field distribution of T_2_ mode (**e**) and T_5_ mode (**f**) in x-z plane (y=0) were shown.

1. **The simulated sensing performance of nanohole arrays with different hole diameters**

The transmission spectra of the nanohole arrays with D=280 nm, 350 nm and 440 nm were simulated in different media as the background refractive index ranging from 1 to 1.3786. Fig. S2 shows the different evolution of the spectra of the GNAs with different diameters.

**
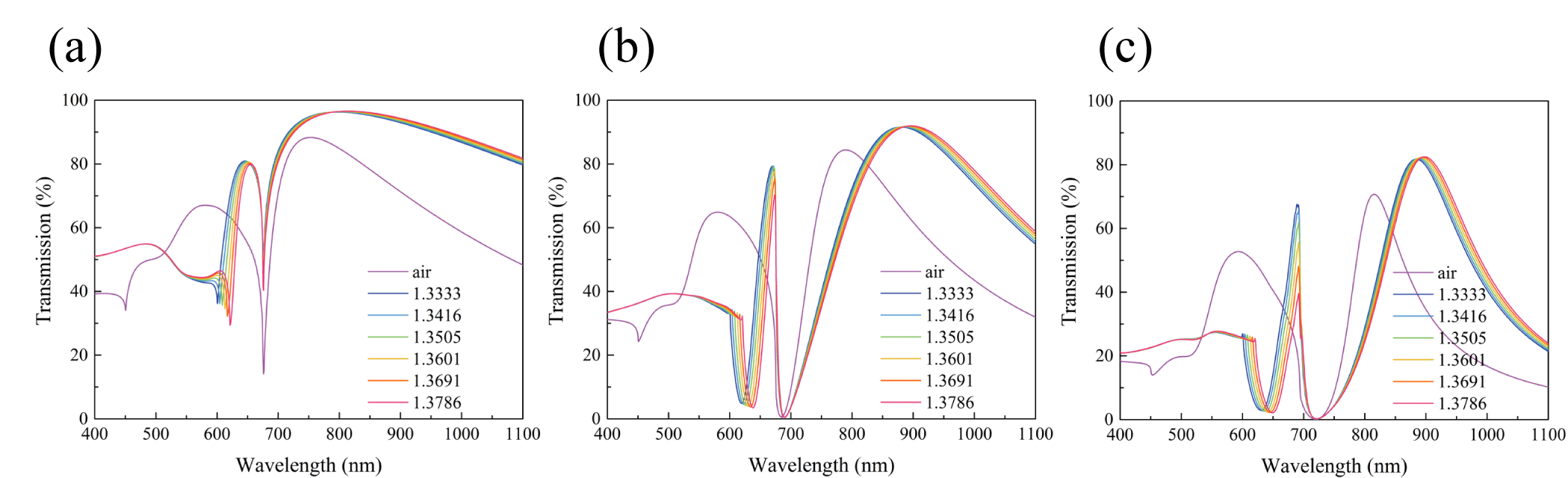
Fig. S2.** Simulated spectra of nanohole arrays (P=520nm) with different hole diameters to evaluate their sensing performance. The transmission spectra of nanohole arrays with diameter of (**a**) 440 nm, (**b**) 350 nm and (**c**) 280 nm in air and different background refractive index from 1.3333 to 1.3786.

1. **The biosensing of BSA molecules with GNAs**

The transmission spectra of the nanohole arrays in BSA solutions with different concentrations were shown in the Fig. S3a. In order to extract the dip wavelength position accurately, we applied a third-order polynomial fitting to the original spectral dip and obtained the resonance wavelength from the fitting curve(Fig. S3b).


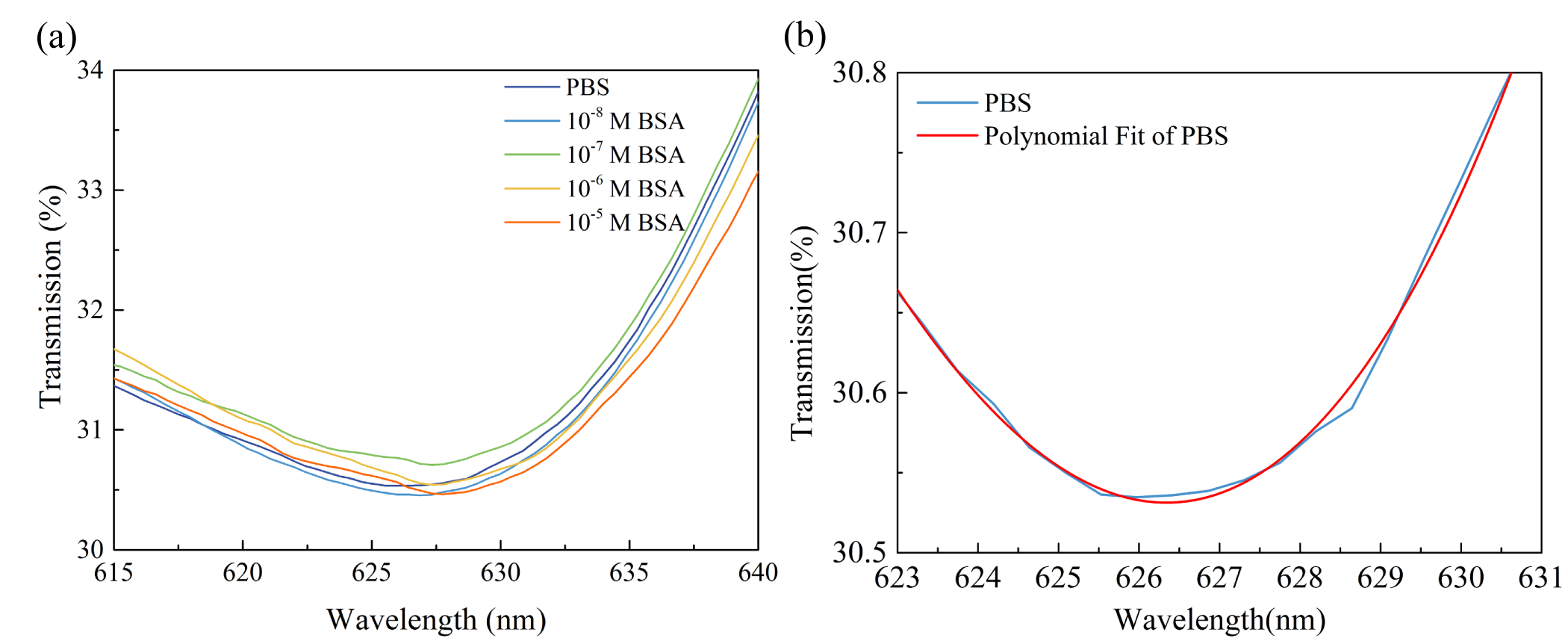


**Figure S3.** (**a**)The original transmission spectra of the GNA (D=440nm) in PBS solutions and BSA solutions with different concentrations (10^-8^ M to 10^-5^ M). (**b**) The original experimental data in PBS solution and the processed data with a third-order polynomial fitting method.

1. **Data extraction and processing from the smartphone camera**

The images collected by the smartphone camera were processed in a MATLAB program and the intensity data in the red (R) channel were extracted. Fig. S4a and S4b show the original and replotted image by MATLAB, respectively. The transmitted LED spot is clearly shown in the 331x331 pixel image. We then select the central area (100x100 pixel, the square with white dashed line) shown in Fig. S4b and sum up the intensity values from all the enclosed pixels in the square. For each refractive index, a value is obtained and denoted as Sum_R_n_. For example, Sum_R_1.3333_ is the intensity sum when the GNA is immersed in water and is used as reference. Then difference of relative intensity is calculated with the following equation,

$$Difference of relative intensity= \frac{{Sum}_{R\_n}-{Sum}_{R\_1.3333}}{{Sum}_{R\_1.3333}}\times100\%,$$

where n is the refractive index of the solution.


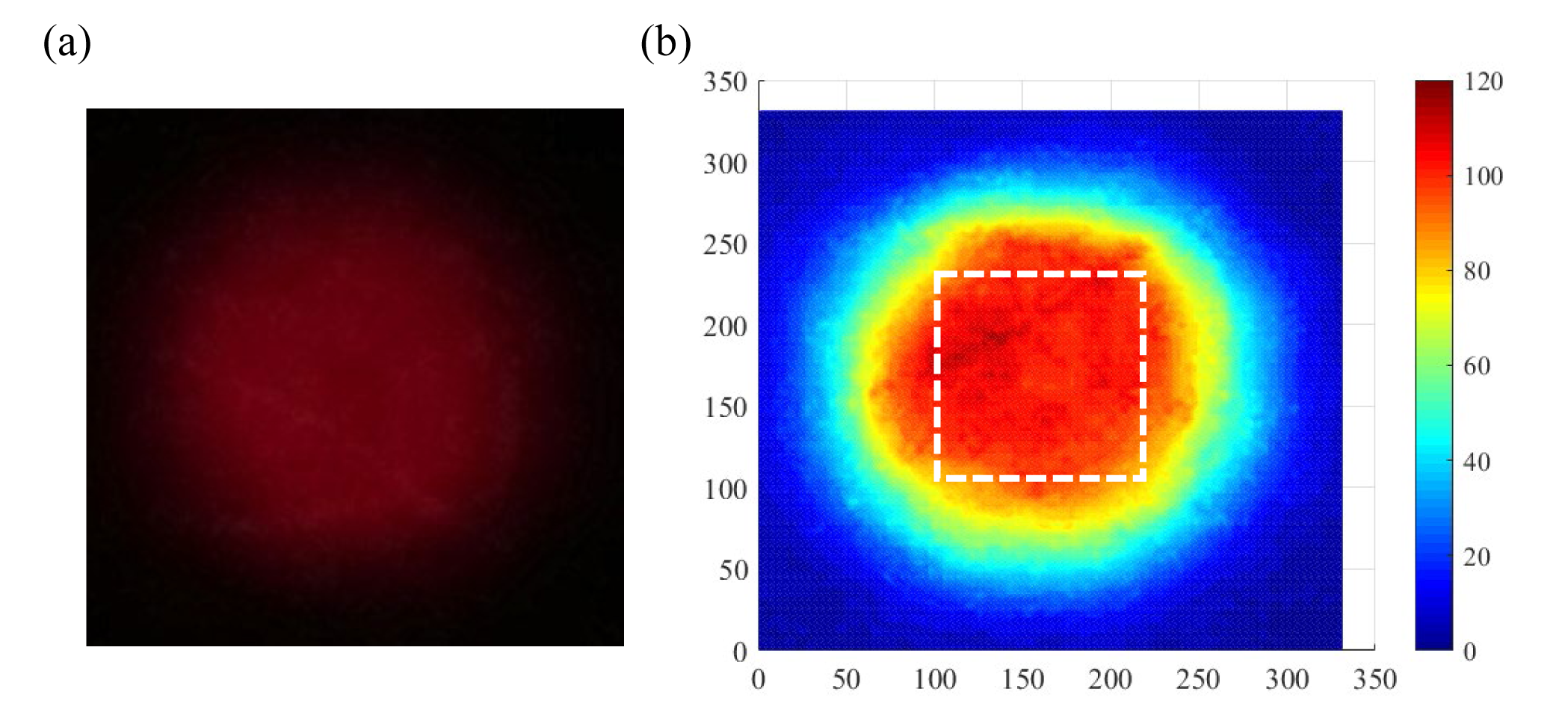


**Figure S4.** (**a**)The original image collected by the smartphone camera. (b) The replotted image in MATLAB showing the intensity data from the red (R) channel of the original image. The area enclosed by the white dashed line is the region-of-interest for our data processing.

[1] H. M. Luong, M. T. Pham, R. P. Madhogaria, M.-H. Phan, G. K. Larsen, T. D. J. N. e. Nguyen, *Nano Energy* **2020**, 71, 104558.

[2] Q. Li, Z. Li, X. Wang, T. Wang, H. Liu, H. Yang, Y. Gong, J. Gao, *Nanoscale* **2018**, 10, 19117.

[3] S. Larson, D. Carlson, B. Ai, Y. Zhao, *Physical Chemistry Chemical Physics* **2019**, 21, 3771.

[4] W. A. Murray, S. Astilean, W. L. Barnes, *Physical Review B* **2004**, 69.

[5] A. Degiron, T. W. Ebbesen, *Journal of Optics a-Pure and Applied Optics* **2005**, 7, S90.

[6] S. H. Chang, S. K. Gray, G. C. Schatz, *Optics Express* **2005**, 13, 3150.
